# Crystal structures of three functionalized chalcones: 4′-di­methyl­amino-3-nitro­chalcone, 3-di­methyl­amino-3′-nitrochalcone and 3′-nitro­chalcone

**DOI:** 10.1107/S2056989020011858

**Published:** 2020-09-04

**Authors:** Charlie L. Hall, Victoria Hamilton, Jason Potticary, Matthew E. Cremeens, Natalie E. Pridmore, Hazel A. Sparkes, Gemma D. D’ambruoso, Stephen D. Warren, Masaomi Matsumoto, Simon R. Hall

**Affiliations:** a School of Chemistry, University of Bristol, Cantock’s Close, Bristol, BS8 1TS, England; b Department of Chemistry & Biochemistry, Gonzaga University, 502 E Boone Ave, Spokane, WA 99258, USA

**Keywords:** chalcone, di­methyl­amino, nitro, crystal structure

## Abstract

The structure of three functionalized chalcones (1,3-di­aryl­prop-2-en-1-ones), containing combinations of nitro and di­methyl­amino functional groups, are presented, namely, 1-[4-(di­methyl­amino)­phen­yl]-3-(3-nitro­phen­yl)prop-2-en-1-one, C_17_H_16_N_2_O_3_, Gp8m, 3-[3-(di­methyl­amino)­phen­yl]-1-(3-nitro­phen­yl)prop-2-en-1-one, C_17_H_16_N_2_O_3_, Hm7m and 1-(3-nitro­phen­yl)-3-phenyl­prop-2-en-1-one, C_15_H_11_NO_3_, Hm1-. Each of the mol­ecules contains bonding motifs seen in previously solved crystal structures of functionalized chalcones, adding to the large dataset available for these small organic mol­ecules. The structures of all three of the title compounds contain similar bonding motifs, resulting in two-dimensional planes of mol­ecules formed *via* C—H⋯O hydrogen-bonding inter­actions involving the nitro- and ketone groups. The structure of Hm1- is very similar to the crystal structure of a previously solved isomer [Jing (2009 ▸). *Acta Cryst.* E**65**, o2510].

## Abstract 2

The structure of three functionalized chalcones (1,3-di­aryl­prop-2-en-1-ones), containing combinations of nitro and di­methyl­amino functional groups, are presented.

## Chemical context   

Chalcones, 1,3-di­aryl­prop-2-en-1-ones, are a group of organic mol­ecules containing two aromatic rings joined by an enone backbone. Chalcones are studied for a range of medicinal purposes, with many reviews published on their biological applicability (see, for example, Rammohan *et al.*, 2020 ▸; Zhuang *et al.*, 2017 ▸; Singh *et al.*, 2014 ▸).

A range of chalcones, functionalized on either aromatic ring, can be readily synthesized *via* an aldol condensation reaction (Mandge *et al.*, 2007 ▸). Altering the functional groups on the chalcone structure has been shown to yield a variety of useful properties, including changes in colour and fluorescent properties (Ibnaouf *et al.*, 2018 ▸).
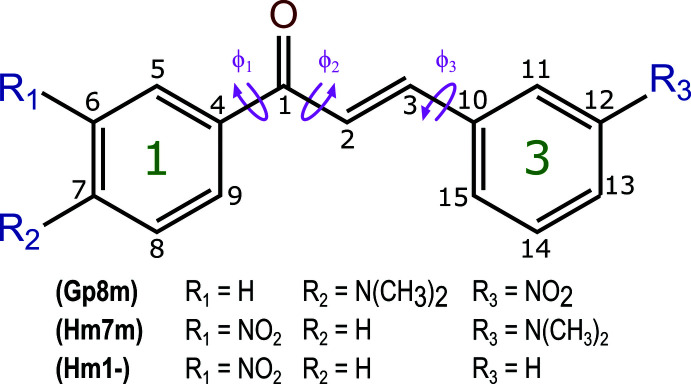


In this work, the structures of three chalcones: 4′-di­methyl­amino-3-nitro­chalcone [Gp8m, *R*_1_ = N(CH_3_)_2_, *R*_2_ = H, *R*_3_ = NO_2_], 3′-nitro-3-di­methyl­amino­chalcone [Hm7m, *R*_1_ = NO_2_, *R*_2_ = N(CH_3_)_2_, *R*_3_ = H] and 3′-nitro­chalcone [Hm1-, *R*_1_ = NO_2_, *R*_2_ = H, *R*_3_ = H] are presented. The crystal structures of these mol­ecules add to the large dataset available for mol­ecules based on the chalcone backbone. In particular, these structures add to the small amount of data available for chalcones substituted with a nitro group on the 3-ring.

## Structural commentary   

The planarity of the chalcone mol­ecules is defined by the torsion angles Φ_1_ = C5—C4—C1—C2, Φ_2_ = C4—C1—C2—C3 and Φ_3_ = C2—C3—C10—C11. The torsion angle C1—C2—C3—C10 is planar to within 1° of 180° in all three structures. The torsion angles, along with the numbering of the mol­ecules, are highlighted in the scheme. The 1-ring of the mol­ecule is defined as the aromatic ring attached to C1 and the 3-ring is that attached to C3. The long axis of each mol­ecule is defined to be along the C2–C12 axis, and the short axis is defined to be along H2–C2. Table 1 ▸ presents a summary of the torsion and ring angles in the title structures.

**Figure 1:**
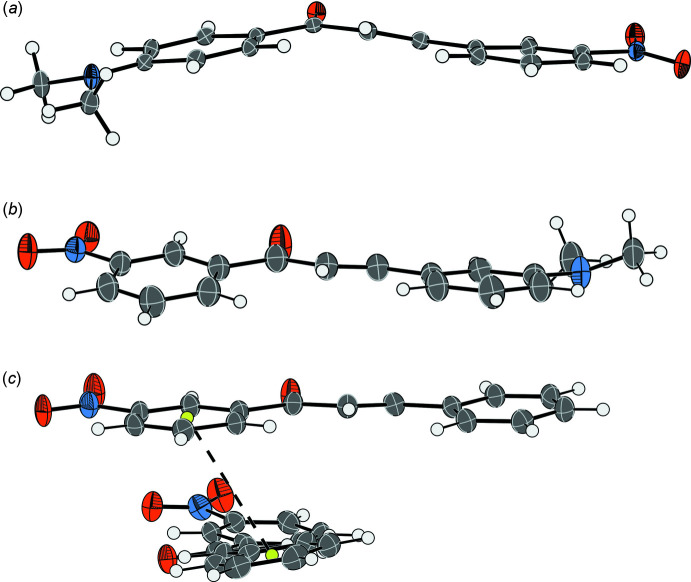
Displacement ellipsoid plots showing the asymmetric units of the solved crystal structures (*a*) Gp8m, (*b*) Hm7m and (*c*) Hm1-. Displacement ellipsoids are shown at the 50% probability level. The stacking inter­action between the 1- and 3-rings of the mol­ecules in the asymmetric unit of Hm1- is highlighted.

Gp8m (Fig. 1 ▸ *a*) crystallizes in space group *P*2_1_/*c* with a single mol­ecule in the asymmetric unit. The mol­ecule deviates from planarity, with Φ_2_ = 158.84 (12)°, meaning that there is a fold angle of 11.46 (4)° between the planes of the 1- and 3- rings of the mol­ecule. The nitro group on the 3-ring is twisted out of the plane of the ring [C11—C12—N2—O2 = 10.09 (18)°].

Hm7m (Fig. 1 ▸ *b*) crystallizes in space group *P*2_1_/*n* with a single mol­ecule in the asymmetric unit. The combination of torsion angles along the long-axis of the mol­ecule means that although the backbone remains relatively planar [C4—C10—C2 = 2.86 (6)°], the 1- and 3-rings are twisted with respect to each other, with a twist angle of 13.80 (8)° between the planes of the rings.

Hm1- crystallizes in space group *P*2_1_/*c* and contains two mol­ecules in the asymmetric unit. One of the mol­ecules (1) is almost planar, with a twist angle of only 1.88 (8)° between the planes of the 1- and 3-rings of the mol­ecule. The second mol­ecule (2) is less planar with Φ_1_ = −164.6 (2) and Φ_2_ = −172 (9)°, leading to a twist angle of 12.85 (8)° between the planes of the 1- and 3-rings. There is a stacking inter­action between the aryl rings of the two mol­ecules in the asymmetric unit, with a centroid-to-centroid distance of 3.82782 (17) Å (Fig. 1 ▸ *c*).

In each of the mol­ecular structures, the functionalized group in the *meta*-position sits on the same side of the mol­ecule as the carbonyl oxygen group (1-ring: C6, 3-ring: C12). This is likely due to the optimization of hydrogen-bonding motifs in the crystal structures.

## Supra­molecular features   

**Figure 2:**
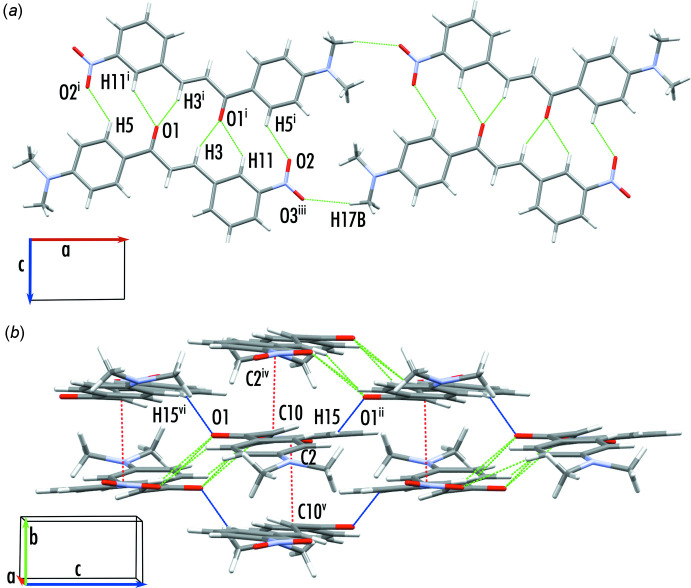
Bonding motifs present in the crystal structure of Gp8m. (*a*) Inter­actions in the plane of the rings of the mol­ecules. (*b*) Hydrogen bonds offset along the short axis of the mol­ecule. Cyclic hydrogen bonds highlighted in (*a*) are shown in green and an additional hydrogen-bonding motif is shown in blue. Stacking inter­actions are highlighted in red. [Symmetry codes: (i) −*x* + 1, −*y* + 1, −*z*; (ii) *x*,-*y* + 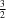, *z* + 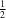; (iii) *x* − 1, *y*, *z*; (iv) −*x* + 1, *y* + 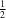, −*z* − 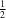; (v) −*x* + 1, *y* + 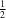, −*z* + 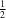; (vi) *x*, −*y* + 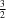, *z* − 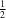.]

Although being in a different space group, the crystal structure of Gp8m is very similar to that of a previously reported chalcone 3′-nitro,4-di­methyl­amino­chalcone (Rosli *et al.*, 2007 ▸). This may be expected, as the only difference between these mol­ecules is that the functional groups are on opposite rings. Within the crystal structure, chains of mol­ecules form down the long axis of the mol­ecule *via* short contacts between the di­methyl­amino and nitro groups (Fig. 2 ▸ *a*; C17—H17*B*⋯O3^iii^). These mol­ecules form stacks parallel to the *b* axis, with alternate mol­ecules the opposite way around such that the nitro group sits above the 1-ring of the adjacent mol­ecule. The final 3D structure is completed by a linking of the stacks *via* C—H⋯O cyclic hydrogen bonding (C3—H3⋯O1^i^, C5—H5⋯O2^i^, C11—H11⋯O1^i^) and hydrogen bonds involving the carbonyl group (Fig. 2 ▸ *b*; C15—H15⋯O1^ii^). Numerical details of the hydrogen-bond geometry and symmetry codes are given in Table 2 ▸.

**Figure 3:**
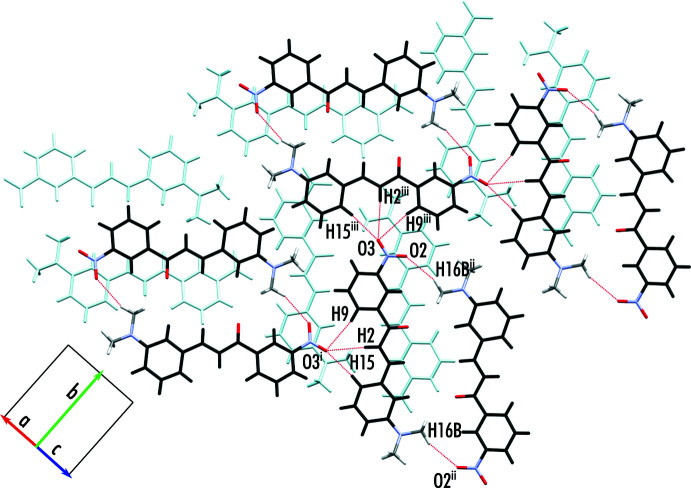
Bonding motifs present in the crystal structure of Hm7m. Two sheets of mol­ecules are highlighted, coloured in black and light blue for contrast. [Symmetry codes: (i) −*x* + 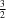, *y* − 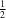, −*z* + 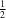; (ii) −*x*, −*y* + 1, −*z* + 1; (iii) −*x* + 1, −*y* + 1, −*z* + 1.]

Within the crystal structure of Hm7m, sheets are formed in the plane of the aromatic rings of the mol­ecule. Within the plane, pairs of inverted mol­ecules form *via* cyclic hydrogen bonding between the di­methyl­amino and nitro groups, offset in the short axis of the mol­ecule (C16—H16*B*⋯O2^ii^). The pairs of mol­ecules then form sheets *via* a trifurcated hydrogen-bonding inter­action involving the nitro group (C15—H15⋯O3^i^, C15—H2⋯O3^i^, C15—H9⋯O3^i^). These sheets make up the 3D structure *via* a stacking inter­action, where the nitro group of one mol­ecule sits over the 1- ring of another (Fig. 3 ▸). Numerical details of the hydrogen-bond geometry and symmetry codes are given in Table 3 ▸.

**Figure 4:**
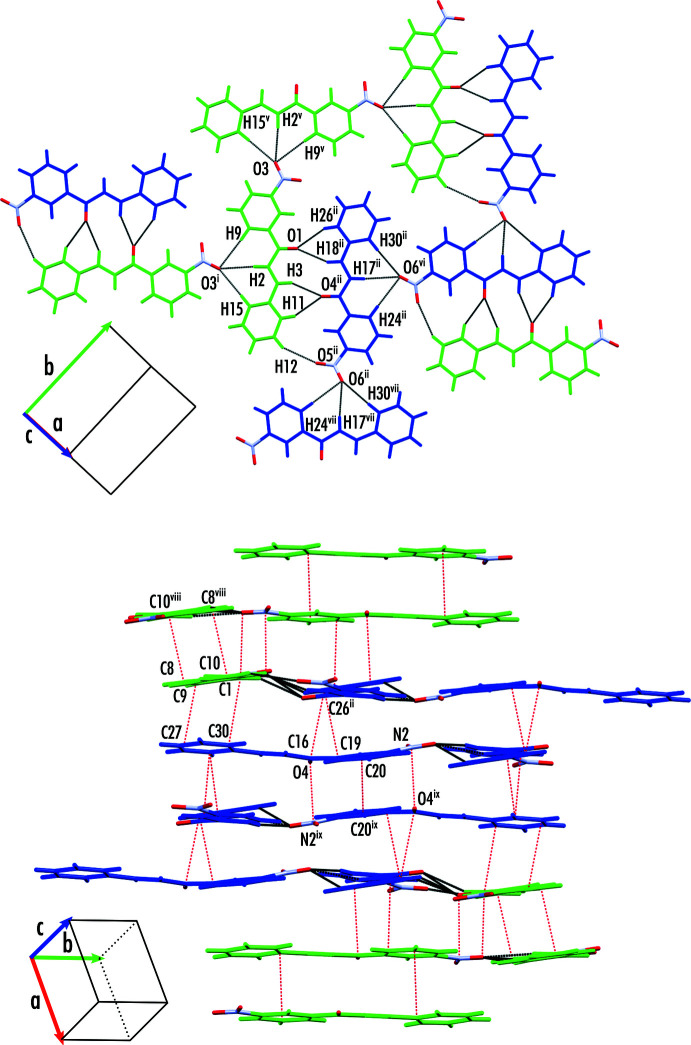
Bonding motifs present in the crystal structure of Hm1-. (*a*) Hydrogen-bonding inter­actions forming two-dimensional planes of mol­ecules. (*b*) Stacking inter­actions present in the crystal structure. Mol­ecules are coloured according to their symmetry equivalence as green (1) and blue (2). [Symmetry codes: (i) −*x*, *y* − 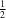, −*z* + 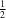; (ii) *x*, −*y* + 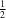, *z* + 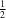; (v) −*x*, *y* + 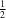, −*z* + 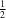; (vi) −*x* + 1, −*y* + 1, −*z* + 2; (vii) −*x* + 1, −*y*, −*z* + 2; (viii) −*x*, −*y*, −*z* + 1; (ix) −*x* + 1, −*y* + 1, −*z* + 1.]

The crystal structure of Hm1- contains two mol­ecules in the asymmetric unit cell, which differ slightly in their planarity. Sheets of mol­ecules form *via* the same inter­actions as in Hm7m; however, the pairs of mol­ecules form between different independent mol­ecules, meaning they are not directly related by an inversion centre. Furthermore, the absence of the di­methyl­amino group means that the mol­ecules are shifted relatively along the long axis of the mol­ecule, forming hydrogen bonds that utilize the carbonyl oxygen (Fig. 4 ▸ *a*). The stacking inter­actions that make up the 3D structure of Hm1- are more complex than those in Hm7m, and are highlighted in Fig. 4 ▸ *b*. Mol­ecule 1 forms a direct stack with a symmetrically equivalent mol­ecule, with an inversion centre relating the mol­ecules. There is a half stack that forms between the 1-ring of mol­ecule 1 and the 3-ring of mol­ecule 2, which sit at approximately 90° to each other. Finally, mol­ecule 2 forms a half stack with a symmetrically equivalent mol­ecule, where the 1-ring of each mol­ecule sits on top of the other. Numerical details of the hydrogen-bond geometry are given in Table 4 ▸.

## Database survey   

A survey of the Cambridge Structural Database (CSD, version 5.41, last update March 2020; Groom *et al.*, 2016 ▸) revealed 38 structures of chalcones functionalized with either nitro or di­methyl­amino-groups in either the *meta* or *para* positions of the 1- or 3-ring. None of the structures contain chalcones with a di­methyl­amino group on the 1- ring, as in Gp8m. However, there are 14 structures of chalcones substituted with a di­methyl­amino group on the 3-ring, likely owing to their fluorescent properties (Jiang *et al.*, 1994 ▸; Tomasch *et al.*, 2012 ▸).

**Figure 5:**
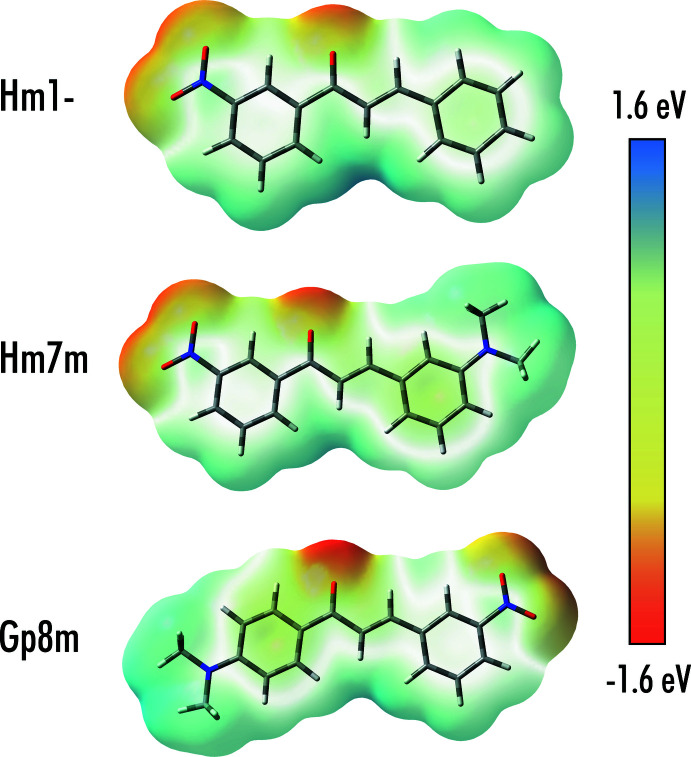
Electrostatic potential maps for optimized configurations of Gp8m, Hm7m and Hm1- mol­ecules. Optimizations were carried out using *Gaussian09* [B3LYP/6–31 G(*d*); Frisch *et al.*, 2009 ▸]. The electrostatic potential is mapped onto the 0.0004 SCF electron density surface.

17 of the 29 structures that contain nitro ring substitutions contain the bonding motif between the nitro group and the region between H15, H2 and H9, as observed in Hm7m and Hm1-. This is likely caused by the optimization of electrostatic inter­actions, as highlighted by the electrostatic potentials in Fig. 5 ▸. The layered motif in Hm7m is the same as that present in the structure of 3′-nitro-3,5-di­meth­oxy­chalcone (Qiu & Yang, 2006 ▸). The planes of mol­ecules seen in Hm1- are similar to those seen in the structure of 4′-nitro­chalcone (BUDXOO; Jing, 2009 ▸).

## Synthesis and crystallization   

Each of the functionalized chalcones was synthesized *via* an aldol condensation reaction between a suitably functionalized benzaldehyde and aceto­phenone. While syntheses were not specifically reported for Gp8m and Hm7m, the first reports for Hm1- appeared in 1929 and 1935 (Dilthey *et al.*, 1929 ▸; Weygand *et al.*, 1935 ▸).

Ethanol (1.5 mL, 95%) and a stir bar were added to two separate vessels; one contained the benzaldehyde (3 mmol) and the other contained the aceto­phenone (3 mmol). Each vessel was gently heated over a hot plate until complete dissolution and then cooled to room temperature; depending on the solubility of the starting materials, solids precipitated upon cooling. Once cooled, NaOH (aq) (0.4 mL, 50% by wgt) was added to the vessel containing the aceto­phenone and vigorously stirred. The benzaldehyde mixture was added to the aceto­phenone and NaOH mixture. The resulting reaction mixture was vigorously mixed until a slurry or paste formed. Water was added to the vessel and the contents were agitated with a micro spatula. The solids were collected by vacuum filtration and purified by recrystallization with ethanol. ^1^H NMR (400 MHz, CDCl_3_, referenced to TMS): δ (ppm) for Gp8m are 8.51 (1H, *s*), 8.22 (1H, *d*, *J* = 8.0 Hz), 8.02 (2H, *d*, *J* = 8.9 Hz), 7.90 (1H, *d*, *J* = 7.5 Hz), 7.79 (1H, *d*, *J* = 15.6 Hz), 7.70 (1H, *d*, *J* = 15.6 Hz), 7.59 (1H, *t*, *J* = 8.0 Hz), 6.72 (2H, *d*, *J* = 8.9 Hz), 3.11 (6H, *s*); for Hm1- are 8.83 (1H, *t*, *J* = 1.9 Hz), 8.44 (1H, *ddd*, *J* = 8.2, 2.2, 1.0 Hz), 8.35 (1H, *ddd*, *J* = 7.8, 1.4, 1.4 Hz), 7.89 (1H, *d*, *J* = 15.6 Hz), 7.72 (1H, *t*, *J* = 8.0 Hz), 7.67 (2H, *m*), 7.54 (1H, *d*, *J* = 15.6 Hz), 7.45 (3H, *m*); and for Hm7m are 8.82 (1H, *t*, *J* = 1.9 Hz), 8.42 (1H, ddd, J = 8.2, 2.2, 1.0 Hz), 8.34 (1H, ddd, J = 7.7, 1.2, 1.2 Hz), 7.85 (1H, *d*, *J* = 15.6 Hz), 7.71 (1H, *t*, *J* = 8.0 Hz), 7.48 (1H, *d*, *J* = 15.6 Hz), 7.30 (1H, *t*, *J* = 7.9 Hz), 7.06 (1H, *d*, *J* = 7.6 Hz), 6.92 (1H, *dd*, *J* = 2.1, 1.6 Hz), 6.82 (1H, *dd*, *J* = 8.2, 2.5 Hz). ^13^C NMR (100 MHz, CDCl_3_, referenced to solvent, 77.16 ppm): δ (ppm) for Gp8m are 186.86, 153.82, 148.83, 139.52, 137.49, 134.45, 131.16, 130.03, 125.51, 125.05, 124.21, 122.14, 111.02, 40.21; for Hm7m are 188.41, 150.97, 148.49, 148.27, 139.79, 135.06, 134.23, 129.96, 129.79, 127.02, 123.39, 120.42, 116.63, 115.42, 112.82, 40.60; and for Hm1- are 188.09, 148.50, 146.87, 139.58, 134.40, 134.22, 131.33, 130.04, 129.21, 128.86, 127.18, 123.37, 120.72.

Crystals of Hm7m suitable for structural solution *via* single crystal X-ray diffraction were produced *via* evaporation of a 10 mg mL^−1^ acetone solution. Crystals of three separate colours were observed (yellow needles, orange needles and red block-like crystals); however, only crystals of a red block-like morphology were suitable for structure solution. Hm7m appeared to go through a phase transition between 100 K and 200 K which caused the crystal to crack. For this reason, single crystal X-ray diffraction was carried out at 200 K.

Crystals of Hm1- and Gp8m suitable for structural solution *via* single crystal X-ray diffraction were produced *via* evaporation of an ethanol solution of concentration 10 mg mL^−1^. Crystals of Gp8m appeared as fine yellow needles and Hm1- as colourless block-like crystals. Each single crystal was mounted onto a glass capillary using paraffin oil.

## Refinement   

Crystal data, data collection and structure refinement details are summarized in Table 5 ▸. All hydrogen atoms were located geometrically (aromatic C—H = 0.95 Å, methyl C—H = 0.99 Å) and refined using a riding model [*U*_iso_(H) = 1.2*U*_eq_(C-aromatic) or 1.5*U*_eq_(C-meth­yl)].

## Supplementary Material

Crystal structure: contains datablock(s) Hm1-, global, Gp8m, Hm7m. DOI: 10.1107/S2056989020011858/dx2030sup1.cif

Structure factors: contains datablock(s) Gp8m. DOI: 10.1107/S2056989020011858/dx2030Gp8msup2.hkl

Supporting information file. DOI: 10.1107/S2056989020011858/dx2030Gp8msup5.cml

Structure factors: contains datablock(s) Hm7m. DOI: 10.1107/S2056989020011858/dx2030Hm7msup3.hkl

Supporting information file. DOI: 10.1107/S2056989020011858/dx2030Hm7msup6.cml

Structure factors: contains datablock(s) Hm1-. DOI: 10.1107/S2056989020011858/dx2030Hm1-sup4.hkl

Supporting information file. DOI: 10.1107/S2056989020011858/dx2030Hm1-sup7.cml

CCDC references: 2025821, 2025820, 2025819

Additional supporting information:  crystallographic information; 3D view; checkCIF report

## Figures and Tables

**Table 1:** Torsion and ring angles (°) describing the planarity of mol­ecules in each crystal structure Torsion angles calculated using the definitions: Φ_1_ = C5—C4—C1—C2, Φ_2_ = C4—C1—C2—C3 and Φ_3_ = C2—C3—C10—C11. Ring twist and fold angles were calculated using the mean planes of the 1- and 3-rings. All values were calculated using *OLEX2* (Dolomanov *et al.*, 2009 ▸).

	Gm8p	Hm7m	Hm1- (1)	Hm1- (2)
Φ_1_	−173.15 (11)	172.9 (2)	177.4 (2)	−164.6 (2)
Φ_2_	158.84 (12)	168.8 (2)	174.4 (2)	175.9 (2)
Φ_3_	−169.23 (13)	−175.0 (2)	−170.3 (3)	−172.9 (9)
Ring twist angle	3.61 (4)	13.8 (8)	1.88 (8)	12.58 (8)
Ring fold angle	11.46 (4)	0.59 (8)	2.58 (8)	6.66 (8)

**Table 2:** Hydrogen-bond geometry (Å, °) for Gp8m[Chem scheme1]

*D*—H⋯*A*	*D*—H	H⋯*A*	*D*⋯*A*	*D*—H⋯*A*
C3—H3⋯O1^i^	0.95	2.47	3.3124 (16)	148
C5—H5⋯O2^i^	0.95	2.67	3.3067 (17)	125
C11—H11⋯O1^i^	0.95	2.88	3.5773 (16)	131
C15—H15⋯O1^ii^	0.95	2.68	3.5435 (16)	152
C17—H17*B*⋯O3^iii^	0.98	2.68	3.5755 (17)	152

Symmetry codes: (i) 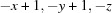; (ii) 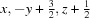; (iii) 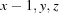.

**Table 3:** Hydrogen-bond geometry (Å, °) for Hm7m[Chem scheme1]

*D*—H⋯*A*	*D*—H	H⋯*A*	*D*⋯*A*	*D*—H⋯*A*
C15—H15⋯O3^i^	0.95	2.50	3.442 (2)	174
C16—H16*B*⋯O2^ii^	0.98	2.58	3.520 (2)	160

Symmetry codes: (i) 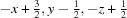; (ii) 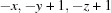.

**Table 4:** Hydrogen-bond geometry (Å, °) for Hm1-[Chem scheme1]

*D*—H⋯*A*	*D*—H	H⋯*A*	*D*⋯*A*	*D*—H⋯*A*
C2—H2⋯O3^i^	0.95	2.51	3.456 (3)	174
C3—H3⋯O4^ii^	0.95	2.50	3.328 (3)	146
C9—H9⋯O3^i^	0.95	2.58	3.527 (3)	175
C15—H15⋯O3^i^	0.95	2.47	3.399 (3)	166
C17—H17⋯O6^iii^	0.95	2.57	3.491 (3)	164
C18—H18⋯O1^iv^	0.95	2.46	3.300 (3)	147
C30—H30⋯O6^iii^	0.95	2.58	3.456 (3)	154
C26—H26⋯O1^iv^	0.95	2.54	3.328 (3)	140

Symmetry codes: (i) 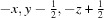; (ii) 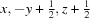; (iii) 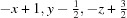; (iv) 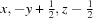.

**Table 5:** Experimental details

	Hm1-	Gp8m	Hm7m
Crystal data			
Chemical formula	C_15_H_11_NO_3_	C_17_H_16_N_2_O_3_	C_17_H_16_N_2_O_3_
*M*_r_	253.25	296.32	296.32
Crystal system, space group	Monoclinic, *P*2_1_/*c*	Monoclinic, *P*2_1_/*c*	Monoclinic, *P*2_1_/*n*
Temperature (K)	100	100	200
*a*, *b*, *c* (Å)	14.7856 (8), 15.9841 (9), 10.3188 (6)	17.3171 (7), 7.0708 (3), 11.3487 (4)	7.7552 (4), 15.6998 (7), 12.0525 (7)
β (°)	99.210 (4)	90.761 (3)	100.668 (3)
*V* (Å^3^)	2407.3 (2)	1389.48 (10)	1442.09 (13)
*Z*	8	4	4
Radiation type	Mo *K*α	Mo *K*α	Mo *K*α
μ (mm^−1^)	0.10	0.10	0.10
Crystal size (mm)	0.61 × 0.35 × 0.25	0.39 × 0.35 × 0.19	0.39 × 0.33 × 0.25

Data collection			
Diffractometer	Bruker APEXII Kappa CCD area detector	Bruker APEXII Kappa CCD area detector	Bruker APEXII Kappa CCD area detector
Absorption correction	Multi-scan (*SADABS*; Bruker, 2016 ▸)	Multi-scan (*SADABS*; Bruker, 2016 ▸)	Multi-scan (*SADABS*; Bruker, 2016 ▸)
*T*_min_, *T*_max_	0.610, 0.746	0.666, 0.746	0.629, 0.746
No. of measured, independent and observed [*I* > 2σ(*I*)] reflections	25080, 4408, 2657	12270, 3320, 2561	11497, 3056, 1882
*R*_int_	0.085	0.034	0.058
(sin θ/λ)_max_ (Å^−1^)	0.602	0.659	0.633

Refinement			
*R*[*F* ^2^ > 2σ(*F* ^2^)], *wR*(*F* ^2^), *S*	0.049, 0.141, 0.99	0.043, 0.123, 1.04	0.049, 0.133, 1.02
No. of reflections	4408	3320	3056
No. of parameters	343	212	201
H-atom treatment	H-atom parameters constrained	H atoms treated by a mixture of independent and constrained refinement	H-atom parameters constrained
Δρ_max_, Δρ_min_ (e Å^−3^)	0.28, −0.25	0.31, −0.28	0.19, −0.24

Computer programs: *APEX2* (Bruker, 2016 ▸), *SAINT* (Bruker, 2016 ▸), *SUPERFLIP* (Palatinus & Chapuis, 2007 ▸; Palatinus & van der Lee, 2008 ▸; Palatinus *et al.*, 2012 ▸), *SHELXL* (Sheldrick, 2015 ▸), *OLEX2* (Dolomanov *et al.*, 2009 ▸) and *Mercury* (Macrae *et al.*, 2020 ▸), *OLEX2* (Dolomanov *et al.*, 2009 ▸).

## supplementary crystallographic information

### 1-[4-(Dimethylamino)phenyl]-3-(3-nitrophenyl)prop-2-en-1-one (Gp8m) Crystal data

**Table:**

C_17_H_16_N_2_O_3_	*F*(000) = 624
*M**_r_* = 296.32	*D*_x_ = 1.416 Mg m^−^^3^
Monoclinic, *P*2_1_/*c*	Mo *K*α radiation, λ = 0.71073 Å
*a* = 17.3171 (7) Å	Cell parameters from 2510 reflections
*b* = 7.0708 (3) Å	θ = 2.4–27.7°
*c* = 11.3487 (4) Å	µ = 0.10 mm^−^^1^
β = 90.761 (3)°	*T* = 100 K
*V* = 1389.48 (10) Å^3^	Plate, clear yellow
*Z* = 4	0.39 × 0.35 × 0.19 mm

### 1-[4-(Dimethylamino)phenyl]-3-(3-nitrophenyl)prop-2-en-1-one (Gp8m) Data collection

**Table:**

Bruker APEXII Kappa CCD area detector diffractometer	3320 independent reflections
Radiation source: fine-focus sealed tube	2561 reflections with *I* > 2σ(*I*)
Graphite monochromator	*R*_int_ = 0.034
φ and ω scans	θ_max_ = 27.9°, θ_min_ = 2.4°
Absorption correction: multi-scan (SADABS; Bruker, 2016)	*h* = −22→22
*T*_min_ = 0.666, *T*_max_ = 0.746	*k* = −9→9
12270 measured reflections	*l* = −14→14

### 1-[4-(Dimethylamino)phenyl]-3-(3-nitrophenyl)prop-2-en-1-one (Gp8m) Refinement

**Table:**

Refinement on *F*^2^	Primary atom site location: iterative
Least-squares matrix: full	Secondary atom site location: difference Fourier map
*R*[*F*^2^ > 2σ(*F*^2^)] = 0.043	Hydrogen site location: mixed
*wR*(*F*^2^) = 0.123	H atoms treated by a mixture of independent and constrained refinement
*S* = 1.04	*w* = 1/[σ^2^(*F*_o_^2^) + (0.0678*P*)^2^ + 0.1379*P*] where *P* = (*F*_o_^2^ + 2*F*_c_^2^)/3
3320 reflections	(Δ/σ)_max_ < 0.001
212 parameters	Δρ_max_ = 0.31 e Å^−^^3^
0 restraints	Δρ_min_ = −0.28 e Å^−^^3^

### 1-[4-(Dimethylamino)phenyl]-3-(3-nitrophenyl)prop-2-en-1-one (Gp8m) Special details

**Table:**

Geometry. All esds (except the esd in the dihedral angle between two l.s. planes) are estimated using the full covariance matrix. The cell esds are taken into account individually in the estimation of esds in distances, angles and torsion angles; correlations between esds in cell parameters are only used when they are defined by crystal symmetry. An approximate (isotropic) treatment of cell esds is used for estimating esds involving l.s. planes.

### 1-[4-(Dimethylamino)phenyl]-3-(3-nitrophenyl)prop-2-en-1-one (Gp8m) Fractional atomic coordinates and isotropic or equivalent isotropic displacement parameters (Å^2^)

**Table:**

	*x*	*y*	*z*	*U*_iso_*/*U*_eq_	
O1	0.40268 (5)	0.64764 (14)	0.01669 (8)	0.0193 (2)	
N1	0.07275 (6)	0.53937 (17)	0.25056 (10)	0.0205 (3)	
C1	0.38550 (7)	0.64612 (17)	0.12161 (11)	0.0145 (3)	
O2	0.81017 (6)	0.60742 (15)	0.17555 (9)	0.0254 (3)	
N2	0.79887 (6)	0.59843 (16)	0.28197 (10)	0.0189 (3)	
C2	0.44638 (7)	0.65714 (18)	0.21487 (12)	0.0162 (3)	
H2	0.432687	0.695454	0.292062	0.019*	
O3	0.85104 (6)	0.57987 (16)	0.35600 (9)	0.0292 (3)	
C3	0.51981 (7)	0.61451 (17)	0.19275 (12)	0.0156 (3)	
H3	0.532011	0.578168	0.114587	0.019*	
C4	0.30435 (7)	0.62866 (17)	0.15869 (11)	0.0141 (3)	
C5	0.24711 (7)	0.59749 (18)	0.07252 (11)	0.0154 (3)	
H5	0.261307	0.595641	−0.008048	0.019*	
C6	0.17102 (8)	0.56948 (18)	0.10089 (12)	0.0168 (3)	
H6	0.133772	0.548627	0.040059	0.020*	
C7	0.14770 (7)	0.57146 (18)	0.22001 (11)	0.0155 (3)	
C8	0.20495 (7)	0.60892 (18)	0.30661 (12)	0.0172 (3)	
H8	0.190843	0.615961	0.387096	0.021*	
C9	0.28085 (7)	0.63541 (18)	0.27630 (12)	0.0163 (3)	
H9	0.318238	0.658831	0.336557	0.020*	
C10	0.58321 (7)	0.61938 (17)	0.27977 (11)	0.0149 (3)	
C11	0.65932 (7)	0.60289 (18)	0.24156 (11)	0.0154 (3)	
H11	0.669921	0.586105	0.160316	0.018*	
C12	0.71904 (7)	0.61135 (17)	0.32371 (12)	0.0159 (3)	
C13	0.70704 (8)	0.62847 (18)	0.44304 (12)	0.0176 (3)	
H13	0.749151	0.632712	0.497489	0.021*	
C14	0.63139 (8)	0.63927 (18)	0.48083 (12)	0.0187 (3)	
H14	0.621331	0.647757	0.562745	0.022*	
C15	0.57042 (8)	0.63789 (18)	0.40118 (12)	0.0174 (3)	
H15	0.519110	0.649624	0.428883	0.021*	
C16	0.01685 (9)	0.4836 (2)	0.16067 (14)	0.0246 (3)	
C17	0.05477 (8)	0.5002 (2)	0.37305 (12)	0.0235 (3)	
H17A	0.083637	0.388553	0.399692	0.035*	
H17B	−0.000728	0.476410	0.380111	0.035*	
H17C	0.069292	0.609223	0.421831	0.035*	
H16A	0.0117 (10)	0.580 (2)	0.0989 (16)	0.033 (5)*	
H16B	0.0326 (10)	0.362 (2)	0.1247 (15)	0.035 (5)*	
H16C	−0.0330 (12)	0.467 (3)	0.1955 (17)	0.049 (5)*	

### 1-[4-(Dimethylamino)phenyl]-3-(3-nitrophenyl)prop-2-en-1-one (Gp8m) Atomic displacement parameters (Å^2^)

**Table:**

	*U*^11^	*U*^22^	*U*^33^	*U*^12^	*U*^13^	*U*^23^
O1	0.0175 (5)	0.0265 (5)	0.0139 (5)	0.0000 (4)	0.0017 (4)	0.0010 (4)
N1	0.0131 (6)	0.0338 (7)	0.0144 (6)	−0.0006 (5)	0.0011 (4)	−0.0003 (5)
C1	0.0165 (7)	0.0124 (6)	0.0147 (6)	0.0013 (5)	0.0006 (5)	−0.0002 (5)
O2	0.0188 (5)	0.0376 (6)	0.0199 (5)	−0.0004 (4)	0.0024 (4)	−0.0025 (4)
N2	0.0158 (6)	0.0198 (6)	0.0210 (6)	0.0003 (4)	−0.0025 (5)	−0.0024 (5)
C2	0.0175 (7)	0.0163 (6)	0.0148 (6)	−0.0001 (5)	−0.0003 (5)	−0.0010 (5)
O3	0.0167 (5)	0.0425 (7)	0.0281 (6)	0.0048 (4)	−0.0078 (4)	−0.0017 (5)
C3	0.0174 (7)	0.0157 (6)	0.0136 (6)	−0.0008 (5)	−0.0001 (5)	0.0003 (5)
C4	0.0154 (6)	0.0121 (6)	0.0147 (7)	0.0008 (5)	−0.0005 (5)	0.0002 (5)
C5	0.0182 (6)	0.0164 (6)	0.0117 (6)	0.0021 (5)	0.0010 (5)	−0.0005 (5)
C6	0.0156 (6)	0.0202 (7)	0.0143 (6)	0.0005 (5)	−0.0037 (5)	−0.0008 (5)
C7	0.0151 (6)	0.0160 (6)	0.0154 (6)	0.0016 (5)	0.0002 (5)	0.0006 (5)
C8	0.0187 (7)	0.0220 (7)	0.0111 (6)	0.0019 (5)	0.0010 (5)	0.0001 (5)
C9	0.0168 (7)	0.0181 (7)	0.0140 (6)	0.0007 (5)	−0.0027 (5)	−0.0009 (5)
C10	0.0163 (6)	0.0127 (6)	0.0157 (7)	0.0001 (5)	−0.0006 (5)	0.0005 (5)
C11	0.0169 (7)	0.0149 (6)	0.0143 (6)	0.0006 (5)	−0.0005 (5)	−0.0003 (5)
C12	0.0152 (6)	0.0145 (6)	0.0181 (7)	0.0018 (5)	−0.0006 (5)	0.0000 (5)
C13	0.0188 (7)	0.0163 (7)	0.0176 (7)	0.0005 (5)	−0.0054 (5)	−0.0004 (5)
C14	0.0254 (7)	0.0185 (7)	0.0122 (6)	−0.0001 (5)	0.0003 (5)	−0.0007 (5)
C15	0.0164 (7)	0.0175 (7)	0.0183 (7)	0.0003 (5)	0.0017 (5)	−0.0009 (5)
C16	0.0148 (7)	0.0382 (9)	0.0207 (8)	−0.0023 (6)	−0.0002 (6)	−0.0016 (7)
C17	0.0168 (7)	0.0359 (8)	0.0181 (7)	0.0008 (6)	0.0046 (5)	0.0051 (6)

### 1-[4-(Dimethylamino)phenyl]-3-(3-nitrophenyl)prop-2-en-1-one (Gp8m) Geometric parameters (Å, º)

**Table:**

O1—C1	1.2312 (15)	C8—H8	0.9500
N1—C7	1.3668 (16)	C8—C9	1.3759 (18)
N1—C16	1.4516 (18)	C9—H9	0.9500
N1—C17	1.4552 (17)	C10—C11	1.3979 (18)
C1—C2	1.4860 (18)	C10—C15	1.4045 (18)
C1—C4	1.4775 (17)	C11—H11	0.9500
O2—N2	1.2277 (15)	C11—C12	1.3845 (18)
N2—O3	1.2326 (14)	C12—C13	1.3781 (18)
N2—C12	1.4700 (17)	C13—H13	0.9500
C2—H2	0.9500	C13—C14	1.3861 (19)
C2—C3	1.3339 (18)	C14—H14	0.9500
C3—H3	0.9500	C14—C15	1.3808 (19)
C3—C10	1.4672 (18)	C15—H15	0.9500
C4—C5	1.4005 (17)	C16—H16A	0.983 (18)
C4—C9	1.4013 (18)	C16—H16B	0.989 (17)
C5—H5	0.9500	C16—H16C	0.96 (2)
C5—C6	1.3749 (18)	C17—H17A	0.9800
C6—H6	0.9500	C17—H17B	0.9800
C6—C7	1.4161 (18)	C17—H17C	0.9800
C7—C8	1.4117 (18)		
			
C7—N1—C16	119.57 (11)	C8—C9—H9	119.2
C7—N1—C17	119.33 (11)	C11—C10—C3	119.30 (12)
C16—N1—C17	118.06 (12)	C11—C10—C15	118.27 (12)
O1—C1—C2	120.67 (11)	C15—C10—C3	122.42 (12)
O1—C1—C4	121.28 (12)	C10—C11—H11	120.4
C4—C1—C2	118.04 (11)	C12—C11—C10	119.11 (12)
O2—N2—O3	123.44 (11)	C12—C11—H11	120.4
O2—N2—C12	118.47 (11)	C11—C12—N2	118.52 (12)
O3—N2—C12	118.09 (11)	C13—C12—N2	118.48 (12)
C1—C2—H2	119.2	C13—C12—C11	122.99 (12)
C3—C2—C1	121.55 (12)	C12—C13—H13	121.2
C3—C2—H2	119.2	C12—C13—C14	117.67 (12)
C2—C3—H3	117.5	C14—C13—H13	121.2
C2—C3—C10	125.07 (12)	C13—C14—H14	119.5
C10—C3—H3	117.5	C15—C14—C13	120.98 (13)
C5—C4—C1	118.77 (11)	C15—C14—H14	119.5
C5—C4—C9	117.30 (12)	C10—C15—H15	119.5
C9—C4—C1	123.90 (12)	C14—C15—C10	120.91 (12)
C4—C5—H5	119.0	C14—C15—H15	119.5
C6—C5—C4	122.05 (12)	N1—C16—H16A	111.4 (10)
C6—C5—H5	119.0	N1—C16—H16B	109.9 (10)
C5—C6—H6	119.7	N1—C16—H16C	109.8 (11)
C5—C6—C7	120.56 (12)	H16A—C16—H16B	109.4 (14)
C7—C6—H6	119.7	H16A—C16—H16C	107.8 (15)
N1—C7—C6	121.62 (12)	H16B—C16—H16C	108.4 (14)
N1—C7—C8	121.00 (12)	N1—C17—H17A	109.5
C8—C7—C6	117.38 (12)	N1—C17—H17B	109.5
C7—C8—H8	119.5	N1—C17—H17C	109.5
C9—C8—C7	121.06 (12)	H17A—C17—H17B	109.5
C9—C8—H8	119.5	H17A—C17—H17C	109.5
C4—C9—H9	119.2	H17B—C17—H17C	109.5
C8—C9—C4	121.59 (12)		
			
O1—C1—C2—C3	−19.7 (2)	C4—C5—C6—C7	0.1 (2)
O1—C1—C4—C5	5.38 (18)	C5—C4—C9—C8	1.23 (19)
O1—C1—C4—C9	−176.74 (12)	C5—C6—C7—N1	−178.37 (12)
N1—C7—C8—C9	177.95 (12)	C5—C6—C7—C8	1.93 (19)
C1—C2—C3—C10	−179.22 (11)	C6—C7—C8—C9	−2.35 (19)
C1—C4—C5—C6	176.37 (12)	C7—C8—C9—C4	0.8 (2)
C1—C4—C9—C8	−176.68 (12)	C9—C4—C5—C6	−1.66 (19)
O2—N2—C12—C11	10.09 (18)	C10—C11—C12—N2	−178.54 (11)
O2—N2—C12—C13	−170.87 (11)	C10—C11—C12—C13	2.5 (2)
N2—C12—C13—C14	−179.61 (11)	C11—C10—C15—C14	−0.17 (19)
C2—C1—C4—C5	−173.15 (11)	C11—C12—C13—C14	−0.61 (19)
C2—C1—C4—C9	4.74 (18)	C12—C13—C14—C15	−1.66 (19)
C2—C3—C10—C11	−169.23 (13)	C13—C14—C15—C10	2.1 (2)
C2—C3—C10—C15	11.5 (2)	C15—C10—C11—C12	−2.01 (18)
O3—N2—C12—C11	−170.20 (12)	C16—N1—C7—C6	6.14 (19)
O3—N2—C12—C13	8.84 (18)	C16—N1—C7—C8	−174.17 (13)
C3—C10—C11—C12	178.72 (12)	C17—N1—C7—C6	166.08 (12)
C3—C10—C15—C14	179.07 (12)	C17—N1—C7—C8	−14.22 (19)
C4—C1—C2—C3	158.84 (12)		

### 1-[4-(Dimethylamino)phenyl]-3-(3-nitrophenyl)prop-2-en-1-one (Gp8m) Hydrogen-bond geometry (Å, º)

**Table:**

*D*—H···*A*	*D*—H	H···*A*	*D*···*A*	*D*—H···*A*
C3—H3···O1^i^	0.95	2.47	3.3124 (16)	148
C5—H5···O2^i^	0.95	2.67	3.3067 (17)	125
C11—H11···O1^i^	0.95	2.88	3.5773 (16)	131
C15—H15···O1^ii^	0.95	2.68	3.5435 (16)	152
C17—H17*B*···O3^iii^	0.98	2.68	3.5755 (17)	152

Symmetry codes: (i) −*x*+1, −*y*+1, −*z*; (ii) *x*, −*y*+3/2, *z*+1/2; (iii) *x*−1, *y*, *z*.

### 3-[3-(Dimethylamino)phenyl]-1-(3-nitrophenyl)prop-2-en-1-one (Hm7m) Crystal data

**Table:**

C_17_H_16_N_2_O_3_	*F*(000) = 624
*M**_r_* = 296.32	*D*_x_ = 1.365 Mg m^−^^3^
Monoclinic, *P*2_1_/*n*	Mo *K*α radiation, λ = 0.71073 Å
*a* = 7.7552 (4) Å	Cell parameters from 1681 reflections
*b* = 15.6998 (7) Å	θ = 2.6–24.0°
*c* = 12.0525 (7) Å	µ = 0.10 mm^−^^1^
β = 100.668 (3)°	*T* = 200 K
*V* = 1442.09 (13) Å^3^	Block, clear orange
*Z* = 4	0.39 × 0.33 × 0.25 mm

### 3-[3-(Dimethylamino)phenyl]-1-(3-nitrophenyl)prop-2-en-1-one (Hm7m) Data collection

**Table:**

Bruker APEXII Kappa CCD area detector diffractometer	3056 independent reflections
Radiation source: fine-focus sealed tube	1882 reflections with *I* > 2σ(*I*)
Graphite monochromator	*R*_int_ = 0.058
φ and ω scans	θ_max_ = 26.7°, θ_min_ = 2.2°
Absorption correction: multi-scan (SADABS; Bruker, 2016)	*h* = −7→9
*T*_min_ = 0.629, *T*_max_ = 0.746	*k* = −19→19
11497 measured reflections	*l* = −15→13

### 3-[3-(Dimethylamino)phenyl]-1-(3-nitrophenyl)prop-2-en-1-one (Hm7m) Refinement

**Table:**

Refinement on *F*^2^	Primary atom site location: iterative
Least-squares matrix: full	Secondary atom site location: difference Fourier map
*R*[*F*^2^ > 2σ(*F*^2^)] = 0.049	Hydrogen site location: inferred from neighbouring sites
*wR*(*F*^2^) = 0.133	H-atom parameters constrained
*S* = 1.02	*w* = 1/[σ^2^(*F*_o_^2^) + (0.0622*P*)^2^ + 0.0748*P*] where *P* = (*F*_o_^2^ + 2*F*_c_^2^)/3
3056 reflections	(Δ/σ)_max_ < 0.001
201 parameters	Δρ_max_ = 0.19 e Å^−^^3^
0 restraints	Δρ_min_ = −0.24 e Å^−^^3^

### 3-[3-(Dimethylamino)phenyl]-1-(3-nitrophenyl)prop-2-en-1-one (Hm7m) Special details

**Table:**

Geometry. All esds (except the esd in the dihedral angle between two l.s. planes) are estimated using the full covariance matrix. The cell esds are taken into account individually in the estimation of esds in distances, angles and torsion angles; correlations between esds in cell parameters are only used when they are defined by crystal symmetry. An approximate (isotropic) treatment of cell esds is used for estimating esds involving l.s. planes.

### 3-[3-(Dimethylamino)phenyl]-1-(3-nitrophenyl)prop-2-en-1-one (Hm7m) Fractional atomic coordinates and isotropic or equivalent isotropic displacement parameters (Å^2^)

**Table:**

	*x*	*y*	*z*	*U*_iso_*/*U*_eq_	
C1	0.3894 (3)	0.47781 (12)	0.36738 (19)	0.0361 (5)	
N1	0.7377 (2)	0.69758 (11)	0.21902 (15)	0.0364 (4)	
O1	0.2877 (2)	0.53451 (9)	0.38190 (16)	0.0554 (5)	
C2	0.3564 (3)	0.38878 (12)	0.39197 (18)	0.0356 (5)	
H2	0.427006	0.345535	0.367975	0.043*	
N2	−0.1631 (2)	0.18000 (10)	0.60898 (17)	0.0411 (5)	
O2	0.62970 (19)	0.74937 (9)	0.23772 (14)	0.0445 (4)	
C3	0.2296 (3)	0.36699 (12)	0.44729 (18)	0.0343 (5)	
H3	0.162525	0.412295	0.469835	0.041*	
O3	0.8619 (2)	0.71586 (10)	0.17312 (14)	0.0518 (5)	
C4	0.5534 (3)	0.50134 (12)	0.32408 (18)	0.0316 (5)	
C5	0.5701 (3)	0.58465 (12)	0.28981 (17)	0.0315 (5)	
H5	0.478821	0.624712	0.291478	0.038*	
C6	0.7212 (3)	0.60857 (12)	0.25325 (18)	0.0320 (5)	
C7	0.8569 (3)	0.55285 (13)	0.24965 (19)	0.0388 (6)	
H7	0.960216	0.571074	0.224810	0.047*	
C8	0.8390 (3)	0.47026 (13)	0.2829 (2)	0.0431 (6)	
H8	0.930866	0.430648	0.280479	0.052*	
C9	0.6885 (3)	0.44375 (13)	0.32009 (19)	0.0386 (5)	
H9	0.677876	0.386332	0.342771	0.046*	
C10	0.1823 (3)	0.28126 (12)	0.47704 (18)	0.0311 (5)	
C11	0.0358 (2)	0.27097 (12)	0.52753 (17)	0.0315 (5)	
H11	−0.028145	0.319845	0.542776	0.038*	
C12	−0.0194 (3)	0.19012 (12)	0.55642 (18)	0.0326 (5)	
C13	0.0783 (3)	0.12007 (13)	0.5326 (2)	0.0406 (6)	
H13	0.044151	0.064351	0.550403	0.049*	
C14	0.2239 (3)	0.13081 (13)	0.4835 (2)	0.0441 (6)	
H14	0.289136	0.082183	0.468976	0.053*	
C15	0.2770 (3)	0.21003 (13)	0.4552 (2)	0.0395 (6)	
H15	0.377263	0.216077	0.421012	0.047*	
C16	−0.2649 (3)	0.25448 (14)	0.6266 (2)	0.0482 (6)	
H16A	−0.187783	0.297063	0.669840	0.072*	
H16B	−0.356654	0.238239	0.668615	0.072*	
H16C	−0.319124	0.278526	0.553447	0.072*	
C17	−0.2612 (3)	0.10108 (14)	0.5915 (2)	0.0464 (6)	
H17A	−0.304660	0.092786	0.510518	0.070*	
H17B	−0.360675	0.103638	0.631031	0.070*	
H17C	−0.184628	0.053444	0.620960	0.070*	

### 3-[3-(Dimethylamino)phenyl]-1-(3-nitrophenyl)prop-2-en-1-one (Hm7m) Atomic displacement parameters (Å^2^)

**Table:**

	*U*^11^	*U*^22^	*U*^33^	*U*^12^	*U*^13^	*U*^23^
C1	0.0362 (12)	0.0342 (12)	0.0402 (14)	−0.0013 (9)	0.0131 (11)	−0.0013 (9)
N1	0.0346 (10)	0.0413 (10)	0.0350 (12)	−0.0075 (8)	0.0110 (9)	0.0033 (8)
O1	0.0486 (10)	0.0365 (9)	0.0918 (15)	0.0039 (7)	0.0405 (10)	0.0111 (8)
C2	0.0374 (12)	0.0329 (11)	0.0389 (14)	−0.0031 (9)	0.0133 (11)	−0.0015 (9)
N2	0.0430 (11)	0.0332 (10)	0.0531 (13)	−0.0082 (8)	0.0248 (10)	−0.0013 (9)
O2	0.0434 (9)	0.0373 (9)	0.0564 (12)	0.0024 (7)	0.0188 (8)	0.0063 (7)
C3	0.0348 (11)	0.0319 (11)	0.0382 (14)	−0.0029 (8)	0.0115 (10)	−0.0020 (9)
O3	0.0476 (10)	0.0526 (10)	0.0637 (13)	−0.0116 (7)	0.0324 (9)	0.0073 (8)
C4	0.0325 (11)	0.0301 (11)	0.0340 (13)	−0.0026 (8)	0.0111 (10)	−0.0020 (9)
C5	0.0298 (11)	0.0327 (11)	0.0338 (13)	−0.0015 (8)	0.0108 (10)	−0.0014 (9)
C6	0.0330 (11)	0.0335 (11)	0.0310 (12)	−0.0053 (9)	0.0096 (10)	−0.0002 (9)
C7	0.0328 (11)	0.0441 (13)	0.0434 (15)	−0.0037 (9)	0.0175 (11)	−0.0006 (10)
C8	0.0353 (12)	0.0400 (13)	0.0578 (17)	0.0046 (9)	0.0189 (12)	−0.0017 (11)
C9	0.0411 (12)	0.0322 (12)	0.0455 (15)	0.0002 (9)	0.0159 (11)	0.0003 (10)
C10	0.0306 (10)	0.0314 (11)	0.0322 (13)	−0.0052 (8)	0.0077 (10)	−0.0016 (9)
C11	0.0319 (11)	0.0306 (11)	0.0330 (13)	0.0008 (8)	0.0090 (10)	−0.0029 (9)
C12	0.0351 (11)	0.0329 (11)	0.0308 (13)	−0.0049 (9)	0.0084 (10)	−0.0017 (9)
C13	0.0452 (13)	0.0274 (11)	0.0513 (16)	−0.0060 (9)	0.0145 (12)	−0.0011 (10)
C14	0.0441 (13)	0.0310 (11)	0.0615 (17)	0.0015 (9)	0.0211 (13)	−0.0060 (11)
C15	0.0371 (12)	0.0376 (12)	0.0481 (15)	−0.0031 (9)	0.0191 (11)	−0.0037 (10)
C16	0.0449 (13)	0.0449 (14)	0.0622 (18)	−0.0052 (10)	0.0292 (13)	−0.0033 (11)
C17	0.0441 (13)	0.0441 (13)	0.0537 (17)	−0.0120 (10)	0.0160 (12)	0.0068 (11)

### 3-[3-(Dimethylamino)phenyl]-1-(3-nitrophenyl)prop-2-en-1-one (Hm7m) Geometric parameters (Å, º)

**Table:**

C1—O1	1.223 (2)	C8—H8	0.9500
C1—C2	1.461 (3)	C8—C9	1.389 (3)
C1—C4	1.507 (3)	C9—H9	0.9500
N1—O2	1.218 (2)	C10—C11	1.394 (2)
N1—O3	1.2303 (19)	C10—C15	1.389 (3)
N1—C6	1.469 (3)	C11—H11	0.9500
C2—H2	0.9500	C11—C12	1.404 (3)
C2—C3	1.331 (3)	C12—C13	1.395 (3)
N2—C12	1.389 (2)	C13—H13	0.9500
N2—C16	1.448 (3)	C13—C14	1.378 (3)
N2—C17	1.449 (3)	C14—H14	0.9500
C3—H3	0.9500	C14—C15	1.373 (3)
C3—C10	1.457 (3)	C15—H15	0.9500
C4—C5	1.385 (3)	C16—H16A	0.9800
C4—C9	1.392 (3)	C16—H16B	0.9800
C5—H5	0.9500	C16—H16C	0.9800
C5—C6	1.378 (2)	C17—H17A	0.9800
C6—C7	1.376 (3)	C17—H17B	0.9800
C7—H7	0.9500	C17—H17C	0.9800
C7—C8	1.372 (3)		
			
O1—C1—C2	121.68 (18)	C8—C9—H9	120.0
O1—C1—C4	118.63 (18)	C11—C10—C3	118.49 (16)
C2—C1—C4	119.69 (17)	C15—C10—C3	122.07 (17)
O2—N1—O3	123.37 (17)	C15—C10—C11	119.43 (17)
O2—N1—C6	118.95 (15)	C10—C11—H11	119.2
O3—N1—C6	117.68 (16)	C10—C11—C12	121.59 (17)
C1—C2—H2	119.3	C12—C11—H11	119.2
C3—C2—C1	121.38 (18)	N2—C12—C11	121.59 (17)
C3—C2—H2	119.3	N2—C12—C13	121.05 (17)
C12—N2—C16	118.67 (16)	C13—C12—C11	117.34 (18)
C12—N2—C17	118.37 (16)	C12—C13—H13	119.6
C16—N2—C17	115.23 (17)	C14—C13—C12	120.73 (18)
C2—C3—H3	116.4	C14—C13—H13	119.6
C2—C3—C10	127.12 (18)	C13—C14—H14	119.2
C10—C3—H3	116.4	C15—C14—C13	121.64 (18)
C5—C4—C1	117.81 (16)	C15—C14—H14	119.2
C5—C4—C9	119.36 (17)	C10—C15—H15	120.4
C9—C4—C1	122.81 (18)	C14—C15—C10	119.26 (18)
C4—C5—H5	120.5	C14—C15—H15	120.4
C6—C5—C4	119.01 (17)	N2—C16—H16A	109.5
C6—C5—H5	120.5	N2—C16—H16B	109.5
C5—C6—N1	118.21 (17)	N2—C16—H16C	109.5
C7—C6—N1	119.26 (17)	H16A—C16—H16B	109.5
C7—C6—C5	122.52 (19)	H16A—C16—H16C	109.5
C6—C7—H7	120.9	H16B—C16—H16C	109.5
C8—C7—C6	118.21 (17)	N2—C17—H17A	109.5
C8—C7—H7	120.9	N2—C17—H17B	109.5
C7—C8—H8	119.5	N2—C17—H17C	109.5
C7—C8—C9	120.91 (18)	H17A—C17—H17B	109.5
C9—C8—H8	119.5	H17A—C17—H17C	109.5
C4—C9—H9	120.0	H17B—C17—H17C	109.5
C8—C9—C4	119.98 (19)		
			
C1—C2—C3—C10	179.8 (2)	C4—C5—C6—N1	−179.00 (19)
C1—C4—C5—C6	177.95 (19)	C4—C5—C6—C7	−0.2 (3)
C1—C4—C9—C8	−177.7 (2)	C5—C4—C9—C8	0.6 (3)
N1—C6—C7—C8	179.5 (2)	C5—C6—C7—C8	0.6 (3)
O1—C1—C2—C3	−10.6 (4)	C6—C7—C8—C9	−0.5 (4)
O1—C1—C4—C5	−7.7 (3)	C7—C8—C9—C4	−0.1 (4)
O1—C1—C4—C9	170.6 (2)	C9—C4—C5—C6	−0.4 (3)
C2—C1—C4—C5	172.9 (2)	C10—C11—C12—N2	178.6 (2)
C2—C1—C4—C9	−8.8 (3)	C10—C11—C12—C13	0.0 (3)
C2—C3—C10—C11	−175.0 (2)	C11—C10—C15—C14	0.2 (3)
C2—C3—C10—C15	4.1 (4)	C11—C12—C13—C14	0.5 (3)
N2—C12—C13—C14	−178.0 (2)	C12—C13—C14—C15	−0.8 (4)
O2—N1—C6—C5	9.5 (3)	C13—C14—C15—C10	0.4 (4)
O2—N1—C6—C7	−169.4 (2)	C15—C10—C11—C12	−0.4 (3)
C3—C10—C11—C12	178.7 (2)	C16—N2—C12—C11	4.5 (3)
C3—C10—C15—C14	−178.9 (2)	C16—N2—C12—C13	−177.0 (2)
O3—N1—C6—C5	−170.91 (19)	C17—N2—C12—C11	152.4 (2)
O3—N1—C6—C7	10.2 (3)	C17—N2—C12—C13	−29.1 (3)
C4—C1—C2—C3	168.8 (2)		

### 3-[3-(Dimethylamino)phenyl]-1-(3-nitrophenyl)prop-2-en-1-one (Hm7m) Hydrogen-bond geometry (Å, º)

**Table:**

*D*—H···*A*	*D*—H	H···*A*	*D*···*A*	*D*—H···*A*
C15—H15···O3^i^	0.95	2.50	3.442 (2)	174
C16—H16*B*···O2^ii^	0.98	2.58	3.520 (2)	160

Symmetry codes: (i) −*x*+3/2, *y*−1/2, −*z*+1/2; (ii) −*x*, −*y*+1, −*z*+1.

### 1-(3-Nitrophenyl)-3-phenylprop-2-en-1-one (Hm1-) Crystal data

**Table:**

C_15_H_11_NO_3_	*F*(000) = 1056
*M**_r_* = 253.25	*D*_x_ = 1.398 Mg m^−^^3^
Monoclinic, *P*2_1_/*c*	Mo *K*α radiation, λ = 0.71073 Å
*a* = 14.7856 (8) Å	Cell parameters from 2603 reflections
*b* = 15.9841 (9) Å	θ = 2.4–24.0°
*c* = 10.3188 (6) Å	µ = 0.10 mm^−^^1^
β = 99.210 (4)°	*T* = 100 K
*V* = 2407.3 (2) Å^3^	Block, clear colourless
*Z* = 8	0.61 × 0.35 × 0.25 mm

### 1-(3-Nitrophenyl)-3-phenylprop-2-en-1-one (Hm1-) Data collection

**Table:**

Bruker APEXII Kappa CCD area detector diffractometer	4408 independent reflections
Radiation source: fine-focus sealed tube	2657 reflections with *I* > 2σ(*I*)
Graphite monochromator	*R*_int_ = 0.085
φ and ω scans	θ_max_ = 25.4°, θ_min_ = 1.4°
Absorption correction: multi-scan (SADABS; Bruker, 2016)	*h* = −17→17
*T*_min_ = 0.610, *T*_max_ = 0.746	*k* = −18→19
25080 measured reflections	*l* = −11→12

### 1-(3-Nitrophenyl)-3-phenylprop-2-en-1-one (Hm1-) Refinement

**Table:**

Refinement on *F*^2^	Primary atom site location: iterative
Least-squares matrix: full	Secondary atom site location: difference Fourier map
*R*[*F*^2^ > 2σ(*F*^2^)] = 0.049	Hydrogen site location: inferred from neighbouring sites
*wR*(*F*^2^) = 0.141	H-atom parameters constrained
*S* = 0.99	*w* = 1/[σ^2^(*F*_o_^2^) + (0.0721*P*)^2^] where *P* = (*F*_o_^2^ + 2*F*_c_^2^)/3
4408 reflections	(Δ/σ)_max_ < 0.001
343 parameters	Δρ_max_ = 0.28 e Å^−^^3^
0 restraints	Δρ_min_ = −0.24 e Å^−^^3^

### 1-(3-Nitrophenyl)-3-phenylprop-2-en-1-one (Hm1-) Special details

**Table:**

Geometry. All esds (except the esd in the dihedral angle between two l.s. planes) are estimated using the full covariance matrix. The cell esds are taken into account individually in the estimation of esds in distances, angles and torsion angles; correlations between esds in cell parameters are only used when they are defined by crystal symmetry. An approximate (isotropic) treatment of cell esds is used for estimating esds involving l.s. planes.

### 1-(3-Nitrophenyl)-3-phenylprop-2-en-1-one (Hm1-) Fractional atomic coordinates and isotropic or equivalent isotropic displacement parameters (Å^2^)

**Table:**

	*x*	*y*	*z*	*U*_iso_*/*U*_eq_	
O1	0.15404 (12)	0.16690 (10)	0.61388 (17)	0.0359 (5)	
O2	0.01454 (14)	0.38743 (11)	0.34410 (18)	0.0473 (6)	
O3	−0.05654 (12)	0.36438 (11)	0.14763 (17)	0.0383 (5)	
N1	−0.01292 (15)	0.34002 (13)	0.2521 (2)	0.0323 (5)	
C1	0.12448 (16)	0.11250 (15)	0.5350 (2)	0.0254 (6)	
C2	0.14090 (16)	0.02299 (14)	0.5655 (2)	0.0255 (6)	
H2	0.112881	−0.018632	0.506696	0.031*	
C3	0.19525 (16)	0.00085 (14)	0.6761 (2)	0.0253 (6)	
H3	0.220584	0.045244	0.731502	0.030*	
C4	0.07058 (15)	0.13797 (14)	0.4054 (2)	0.0231 (6)	
C5	0.05461 (16)	0.22346 (14)	0.3858 (2)	0.0248 (6)	
H5	0.077179	0.262547	0.452566	0.030*	
C6	0.00593 (16)	0.25020 (14)	0.2690 (2)	0.0252 (6)	
C7	−0.02844 (17)	0.19594 (15)	0.1684 (2)	0.0279 (6)	
H7	−0.062116	0.216229	0.088457	0.033*	
C8	−0.01236 (16)	0.11190 (15)	0.1879 (2)	0.0271 (6)	
H8	−0.034651	0.073409	0.120088	0.032*	
C9	0.03611 (16)	0.08256 (15)	0.3055 (2)	0.0258 (6)	
H9	0.045864	0.024162	0.317841	0.031*	
C10	0.22033 (16)	−0.08377 (14)	0.7218 (2)	0.0238 (6)	
C11	0.26673 (16)	−0.09467 (15)	0.8493 (2)	0.0266 (6)	
H11	0.282266	−0.047021	0.903293	0.032*	
C12	0.29063 (17)	−0.17356 (15)	0.8988 (2)	0.0301 (6)	
H12	0.321996	−0.179789	0.986089	0.036*	
C13	0.26862 (17)	−0.24322 (16)	0.8206 (3)	0.0325 (6)	
H13	0.284275	−0.297540	0.854305	0.039*	
C14	0.22364 (17)	−0.23354 (15)	0.6929 (3)	0.0302 (6)	
H14	0.209076	−0.281420	0.639024	0.036*	
C15	0.19979 (17)	−0.15487 (15)	0.6432 (2)	0.0282 (6)	
H15	0.169329	−0.148993	0.555349	0.034*	
O4	0.35937 (12)	0.39992 (10)	0.37652 (16)	0.0314 (4)	
O5	0.44124 (12)	0.62138 (10)	0.68540 (17)	0.0357 (5)	
O6	0.52132 (14)	0.59755 (11)	0.87609 (17)	0.0447 (5)	
N2	0.48438 (15)	0.57425 (13)	0.7669 (2)	0.0317 (5)	
C16	0.38593 (16)	0.34642 (15)	0.4587 (2)	0.0261 (6)	
C17	0.36217 (17)	0.25751 (15)	0.4363 (2)	0.0273 (6)	
H17	0.385793	0.216860	0.499959	0.033*	
C18	0.30757 (16)	0.23402 (15)	0.3268 (2)	0.0254 (6)	
H18	0.285045	0.277608	0.267756	0.031*	
C19	0.44235 (15)	0.37308 (14)	0.5864 (2)	0.0231 (5)	
C20	0.44373 (15)	0.45800 (14)	0.6154 (2)	0.0236 (6)	
H20	0.413123	0.496873	0.553799	0.028*	
C21	0.48994 (17)	0.48510 (15)	0.7343 (2)	0.0262 (6)	
C22	0.53737 (16)	0.43117 (16)	0.8256 (2)	0.0294 (6)	
H22	0.568880	0.451388	0.907075	0.035*	
C23	0.53761 (16)	0.34715 (16)	0.7950 (2)	0.0283 (6)	
H23	0.570586	0.309004	0.855604	0.034*	
C24	0.49037 (16)	0.31779 (15)	0.6772 (2)	0.0266 (6)	
H24	0.490620	0.259682	0.657880	0.032*	
C25	0.27816 (15)	0.14936 (15)	0.2866 (2)	0.0243 (6)	
C30	0.29847 (16)	0.07941 (15)	0.3671 (3)	0.0286 (6)	
H30	0.331611	0.086022	0.453284	0.034*	
C29	0.27051 (17)	0.00044 (15)	0.3220 (3)	0.0323 (6)	
H29	0.284069	−0.046713	0.377719	0.039*	
C28	0.22299 (18)	−0.01024 (16)	0.1962 (3)	0.0357 (7)	
H28	0.204939	−0.064705	0.165510	0.043*	
C27	0.20172 (17)	0.05826 (16)	0.1152 (3)	0.0329 (6)	
H27	0.168703	0.051101	0.029069	0.039*	
C26	0.22886 (16)	0.13736 (15)	0.1605 (2)	0.0270 (6)	
H26	0.213718	0.184348	0.104843	0.032*	

### 1-(3-Nitrophenyl)-3-phenylprop-2-en-1-one (Hm1-) Atomic displacement parameters (Å^2^)

**Table:**

	*U*^11^	*U*^22^	*U*^33^	*U*^12^	*U*^13^	*U*^23^
O1	0.0504 (12)	0.0234 (10)	0.0272 (10)	0.0002 (8)	−0.0146 (9)	−0.0021 (8)
O2	0.0765 (15)	0.0291 (11)	0.0296 (11)	0.0078 (10)	−0.0120 (10)	−0.0019 (9)
O3	0.0471 (12)	0.0345 (11)	0.0276 (11)	0.0066 (9)	−0.0113 (9)	0.0096 (8)
N1	0.0386 (13)	0.0309 (12)	0.0249 (13)	0.0054 (10)	−0.0025 (11)	0.0036 (10)
C1	0.0241 (13)	0.0262 (13)	0.0239 (14)	−0.0004 (10)	−0.0020 (11)	0.0002 (11)
C2	0.0295 (14)	0.0218 (13)	0.0237 (14)	−0.0008 (10)	−0.0008 (11)	−0.0023 (10)
C3	0.0298 (14)	0.0219 (13)	0.0230 (14)	−0.0028 (10)	0.0008 (11)	−0.0005 (10)
C4	0.0228 (13)	0.0245 (13)	0.0213 (13)	−0.0006 (10)	0.0011 (11)	0.0032 (11)
C5	0.0289 (14)	0.0236 (14)	0.0207 (13)	−0.0008 (10)	0.0005 (11)	0.0001 (10)
C6	0.0301 (14)	0.0241 (13)	0.0205 (13)	0.0053 (11)	0.0015 (11)	0.0022 (11)
C7	0.0290 (14)	0.0349 (15)	0.0185 (13)	0.0012 (11)	0.0001 (11)	0.0041 (11)
C8	0.0290 (14)	0.0294 (14)	0.0215 (14)	−0.0017 (11)	0.0003 (11)	−0.0023 (11)
C9	0.0275 (13)	0.0259 (14)	0.0230 (14)	0.0001 (10)	0.0010 (11)	0.0013 (11)
C10	0.0245 (13)	0.0245 (13)	0.0213 (13)	−0.0012 (10)	0.0003 (11)	0.0017 (10)
C11	0.0291 (14)	0.0261 (13)	0.0233 (14)	−0.0003 (11)	0.0007 (11)	0.0007 (11)
C12	0.0287 (14)	0.0348 (15)	0.0248 (14)	0.0021 (11)	−0.0024 (12)	0.0061 (12)
C13	0.0365 (15)	0.0248 (14)	0.0358 (16)	0.0039 (11)	0.0047 (13)	0.0062 (12)
C14	0.0348 (15)	0.0255 (14)	0.0299 (15)	−0.0010 (11)	0.0042 (12)	−0.0016 (11)
C15	0.0324 (14)	0.0275 (14)	0.0236 (14)	−0.0006 (11)	0.0015 (12)	0.0021 (11)
O4	0.0389 (10)	0.0259 (9)	0.0255 (10)	−0.0009 (8)	−0.0074 (8)	0.0028 (8)
O5	0.0439 (11)	0.0262 (10)	0.0331 (11)	0.0037 (8)	−0.0057 (9)	−0.0007 (8)
O6	0.0665 (14)	0.0374 (11)	0.0248 (11)	−0.0098 (10)	−0.0090 (10)	−0.0076 (9)
N2	0.0390 (13)	0.0318 (12)	0.0227 (13)	−0.0049 (10)	0.0002 (11)	−0.0043 (10)
C16	0.0252 (13)	0.0274 (14)	0.0256 (14)	0.0022 (11)	0.0034 (11)	−0.0016 (11)
C17	0.0335 (14)	0.0224 (13)	0.0243 (14)	0.0002 (11)	−0.0003 (12)	0.0021 (11)
C18	0.0275 (14)	0.0247 (13)	0.0234 (14)	0.0020 (10)	0.0016 (11)	0.0009 (10)
C19	0.0245 (13)	0.0246 (14)	0.0199 (13)	−0.0026 (10)	0.0027 (11)	0.0005 (10)
C20	0.0258 (13)	0.0236 (13)	0.0207 (13)	−0.0022 (10)	0.0014 (11)	0.0014 (10)
C21	0.0294 (14)	0.0252 (13)	0.0241 (14)	−0.0010 (11)	0.0044 (11)	−0.0006 (11)
C22	0.0296 (14)	0.0366 (15)	0.0206 (14)	−0.0043 (11)	−0.0002 (11)	−0.0002 (11)
C23	0.0249 (13)	0.0316 (15)	0.0269 (15)	0.0014 (11)	−0.0009 (11)	0.0070 (11)
C24	0.0291 (14)	0.0258 (14)	0.0238 (14)	−0.0005 (10)	0.0007 (11)	0.0008 (11)
C25	0.0195 (12)	0.0272 (14)	0.0262 (14)	0.0006 (10)	0.0036 (11)	−0.0035 (11)
C30	0.0272 (13)	0.0274 (14)	0.0300 (15)	0.0014 (11)	0.0004 (11)	−0.0005 (11)
C29	0.0334 (15)	0.0239 (14)	0.0399 (17)	−0.0009 (11)	0.0071 (13)	−0.0014 (12)
C28	0.0363 (16)	0.0263 (15)	0.0450 (18)	−0.0023 (12)	0.0079 (14)	−0.0093 (13)
C27	0.0278 (14)	0.0366 (16)	0.0333 (16)	−0.0029 (11)	0.0022 (12)	−0.0095 (12)
C26	0.0270 (13)	0.0271 (14)	0.0266 (14)	0.0006 (11)	0.0034 (11)	−0.0018 (11)

### 1-(3-Nitrophenyl)-3-phenylprop-2-en-1-one (Hm1-) Geometric parameters (Å, º)

**Table:**

O1—C1	1.223 (3)	O4—C16	1.224 (3)
O2—N1	1.232 (3)	O5—N2	1.229 (3)
O3—N1	1.227 (2)	O6—N2	1.229 (3)
N1—C6	1.468 (3)	N2—C21	1.469 (3)
C1—C2	1.477 (3)	C16—C17	1.473 (3)
C1—C4	1.499 (3)	C16—C19	1.504 (3)
C2—H2	0.9500	C17—H17	0.9500
C2—C3	1.334 (3)	C17—C18	1.333 (3)
C3—H3	0.9500	C18—H18	0.9500
C3—C10	1.460 (3)	C18—C25	1.461 (3)
C4—C5	1.396 (3)	C19—C20	1.389 (3)
C4—C9	1.393 (3)	C19—C24	1.396 (3)
C5—H5	0.9500	C20—H20	0.9500
C5—C6	1.370 (3)	C20—C21	1.375 (3)
C6—C7	1.385 (3)	C21—C22	1.383 (3)
C7—H7	0.9500	C22—H22	0.9500
C7—C8	1.373 (3)	C22—C23	1.380 (3)
C8—H8	0.9500	C23—H23	0.9500
C8—C9	1.388 (3)	C23—C24	1.384 (3)
C9—H9	0.9500	C24—H24	0.9500
C10—C11	1.394 (3)	C25—C30	1.397 (3)
C10—C15	1.401 (3)	C25—C26	1.399 (3)
C11—H11	0.9500	C30—H30	0.9500
C11—C12	1.385 (3)	C30—C29	1.386 (3)
C12—H12	0.9500	C29—H29	0.9500
C12—C13	1.383 (4)	C29—C28	1.384 (4)
C13—H13	0.9500	C28—H28	0.9500
C13—C14	1.387 (4)	C28—C27	1.383 (4)
C14—H14	0.9500	C27—H27	0.9500
C14—C15	1.382 (3)	C27—C26	1.385 (3)
C15—H15	0.9500	C26—H26	0.9500
			
O2—N1—C6	118.5 (2)	O5—N2—O6	123.2 (2)
O3—N1—O2	122.9 (2)	O5—N2—C21	118.7 (2)
O3—N1—C6	118.5 (2)	O6—N2—C21	118.1 (2)
O1—C1—C2	121.2 (2)	O4—C16—C17	121.5 (2)
O1—C1—C4	118.9 (2)	O4—C16—C19	118.7 (2)
C2—C1—C4	119.9 (2)	C17—C16—C19	119.7 (2)
C1—C2—H2	120.1	C16—C17—H17	119.9
C3—C2—C1	119.7 (2)	C18—C17—C16	120.1 (2)
C3—C2—H2	120.1	C18—C17—H17	119.9
C2—C3—H3	116.2	C17—C18—H18	116.1
C2—C3—C10	127.5 (2)	C17—C18—C25	127.9 (2)
C10—C3—H3	116.2	C25—C18—H18	116.1
C5—C4—C1	116.7 (2)	C20—C19—C16	116.9 (2)
C9—C4—C1	124.5 (2)	C20—C19—C24	119.1 (2)
C9—C4—C5	118.8 (2)	C24—C19—C16	124.0 (2)
C4—C5—H5	120.5	C19—C20—H20	120.4
C6—C5—C4	119.1 (2)	C21—C20—C19	119.2 (2)
C6—C5—H5	120.5	C21—C20—H20	120.4
C5—C6—N1	118.2 (2)	C20—C21—N2	118.1 (2)
C5—C6—C7	122.8 (2)	C20—C21—C22	122.5 (2)
C7—C6—N1	118.9 (2)	C22—C21—N2	119.3 (2)
C6—C7—H7	121.0	C21—C22—H22	121.0
C8—C7—C6	118.0 (2)	C23—C22—C21	118.1 (2)
C8—C7—H7	121.0	C23—C22—H22	121.0
C7—C8—H8	119.6	C22—C23—H23	119.6
C7—C8—C9	120.7 (2)	C22—C23—C24	120.8 (2)
C9—C8—H8	119.6	C24—C23—H23	119.6
C4—C9—H9	119.7	C19—C24—H24	119.8
C8—C9—C4	120.6 (2)	C23—C24—C19	120.4 (2)
C8—C9—H9	119.7	C23—C24—H24	119.8
C11—C10—C3	118.8 (2)	C30—C25—C18	123.1 (2)
C11—C10—C15	118.2 (2)	C30—C25—C26	118.3 (2)
C15—C10—C3	123.0 (2)	C26—C25—C18	118.6 (2)
C10—C11—H11	119.3	C25—C30—H30	119.8
C12—C11—C10	121.3 (2)	C29—C30—C25	120.3 (2)
C12—C11—H11	119.3	C29—C30—H30	119.8
C11—C12—H12	120.2	C30—C29—H29	119.8
C13—C12—C11	119.7 (2)	C28—C29—C30	120.5 (2)
C13—C12—H12	120.2	C28—C29—H29	119.8
C12—C13—H13	120.1	C29—C28—H28	120.0
C12—C13—C14	119.8 (2)	C27—C28—C29	120.1 (2)
C14—C13—H13	120.1	C27—C28—H28	120.0
C13—C14—H14	119.7	C28—C27—H27	120.2
C15—C14—C13	120.6 (2)	C28—C27—C26	119.6 (2)
C15—C14—H14	119.7	C26—C27—H27	120.2
C10—C15—H15	119.8	C25—C26—H26	119.4
C14—C15—C10	120.3 (2)	C27—C26—C25	121.2 (2)
C14—C15—H15	119.8	C27—C26—H26	119.4
			
O1—C1—C2—C3	−5.7 (4)	O4—C16—C17—C18	−2.7 (4)
O1—C1—C4—C5	−2.5 (3)	O4—C16—C19—C20	14.0 (3)
O1—C1—C4—C9	177.5 (2)	O4—C16—C19—C24	−167.8 (2)
O2—N1—C6—C5	−1.1 (3)	O5—N2—C21—C20	−2.2 (3)
O2—N1—C6—C7	176.9 (2)	O5—N2—C21—C22	−179.3 (2)
O3—N1—C6—C5	−180.0 (2)	O6—N2—C21—C20	175.9 (2)
O3—N1—C6—C7	−2.0 (3)	O6—N2—C21—C22	−1.1 (3)
N1—C6—C7—C8	−178.0 (2)	N2—C21—C22—C23	176.8 (2)
C1—C2—C3—C10	−179.1 (2)	C16—C17—C18—C25	178.7 (2)
C1—C4—C5—C6	179.8 (2)	C16—C19—C20—C21	176.3 (2)
C1—C4—C9—C8	−179.3 (2)	C16—C19—C24—C23	−177.3 (2)
C2—C1—C4—C5	177.4 (2)	C17—C16—C19—C20	−164.6 (2)
C2—C1—C4—C9	−2.6 (4)	C17—C16—C19—C24	13.6 (4)
C2—C3—C10—C11	−170.3 (2)	C17—C18—C25—C30	5.8 (4)
C2—C3—C10—C15	9.6 (4)	C17—C18—C25—C26	−172.9 (2)
C3—C10—C11—C12	178.7 (2)	C18—C25—C30—C29	−178.4 (2)
C3—C10—C15—C14	−178.6 (2)	C18—C25—C26—C27	177.9 (2)
C4—C1—C2—C3	174.4 (2)	C19—C16—C17—C18	175.9 (2)
C4—C5—C6—N1	177.9 (2)	C19—C20—C21—N2	−175.3 (2)
C4—C5—C6—C7	0.0 (4)	C19—C20—C21—C22	1.7 (4)
C5—C4—C9—C8	0.7 (4)	C20—C19—C24—C23	0.9 (4)
C5—C6—C7—C8	−0.2 (4)	C20—C21—C22—C23	−0.1 (4)
C6—C7—C8—C9	0.7 (4)	C21—C22—C23—C24	−1.1 (4)
C7—C8—C9—C4	−0.9 (4)	C22—C23—C24—C19	0.7 (4)
C9—C4—C5—C6	−0.2 (4)	C24—C19—C20—C21	−2.0 (3)
C10—C11—C12—C13	0.3 (4)	C25—C30—C29—C28	0.6 (4)
C11—C10—C15—C14	1.3 (4)	C30—C25—C26—C27	−0.9 (4)
C11—C12—C13—C14	0.6 (4)	C30—C29—C28—C27	−1.0 (4)
C12—C13—C14—C15	−0.6 (4)	C29—C28—C27—C26	0.5 (4)
C13—C14—C15—C10	−0.4 (4)	C28—C27—C26—C25	0.5 (4)
C15—C10—C11—C12	−1.2 (4)	C26—C25—C30—C29	0.3 (4)

### 1-(3-Nitrophenyl)-3-phenylprop-2-en-1-one (Hm1-) Hydrogen-bond geometry (Å, º)

**Table:**

*D*—H···*A*	*D*—H	H···*A*	*D*···*A*	*D*—H···*A*
C2—H2···O3^i^	0.95	2.51	3.456 (3)	174
C3—H3···O4^ii^	0.95	2.50	3.328 (3)	146
C9—H9···O3^i^	0.95	2.58	3.527 (3)	175
C15—H15···O3^i^	0.95	2.47	3.399 (3)	166
C17—H17···O6^iii^	0.95	2.57	3.491 (3)	164
C18—H18···O1^iv^	0.95	2.46	3.300 (3)	147
C30—H30···O6^iii^	0.95	2.58	3.456 (3)	154
C26—H26···O1^iv^	0.95	2.54	3.328 (3)	140

Symmetry codes: (i) −*x*, *y*−1/2, −*z*+1/2; (ii) *x*, −*y*+1/2, *z*+1/2; (iii) −*x*+1, *y*−1/2, −*z*+3/2; (iv) *x*, −*y*+1/2, *z*−1/2.
